# Biodegradable Polymer Nanosheets Incorporated with Zn-Containing Nanoparticles for Biomedical Applications

**DOI:** 10.3390/ma15228101

**Published:** 2022-11-16

**Authors:** M. Q. Hafzan Ishak, Prabakaran Shankar, Marat E. Turabayev, Takahiro Kondo, Mitsuhiro Honda, Stanislav O. Gurbatov, Yosuke Okamura, Satoru Iwamori, Sergei A. Kulinich

**Affiliations:** 1 Department of Mechanical Engineering, Tokai University, Hiratsuka 259-1292, Japan; 2 Research Institute of Science and Technology, Tokai University, Hiratsuka 259-1292, Japan; 3 Department of Chemistry, Gakushuin University, Toshima-ku, Tokyo 171-0031, Japan; 4 Graduate School of Engineering, Nagoya Institute of Technology, Nagoya 466-8555, Japan; 5 Institute of Automation and Control Processes, Far Eastern Branch, Russian Academy of Science, Vladivostok 690041, Russia; 6 School of Natural Sciences, Far Eastern Federal University, Vladivostok 690091, Russia; 7 Department of Applied Chemistry, Tokai University, Hiratsuka 259-1292, Japan

**Keywords:** biodegradable polymer nanosheets, wound healing, laser ablation in liquid, nanoparticles, ZnO, ZnCl_2_

## Abstract

So far, poly(L-lactic acid), PLLA nanosheets proved to be promising for wound healing. Such biodegradable materials are easy to prepare, bio-friendly, cost-effective, simple to apply and were shown to protect burn wounds and facilitate their healing. At the same time, certain metal ions are known to be essential for wound healing, which is why this study was motivated by the idea of incorporating PLLA nanosheets with Zn^2+^ ion containing nanoparticles. Upon being applied on wound, such polymer nanosheets should release Zn^2+^ ions, which is expected to improve wound healing. The work thus focused on preparing PLLA nanosheets embedded with several kinds of Zn-containing nanoparticles, their characterization and ion-release behavior. ZnCl_2_ and ZnO nanoparticles were chosen because of their different solubility in water, with the intention to see the dynamics of their Zn^2+^ ion release in liquid medium with pH around 7.4. Interestingly, the prepared PLLA nanosheets demonstrated quit similar ion release rates, reaching the maximum concentration after about 10 h. This finding implies that such polymer materials can be promising as they are expected to release ions within several hours after their application on skin.

## 1. Introduction

The treatment of burn wound is known to be a long-lasting and complex process that requires new therapeutic options to improve and accelerate the healing progress [1,2,3,4,5,6,7,8]. An ideal wound dressing should accelerate one or several stages of the healing process including the inflammatory, migratory, proliferative, and remodeling phases. A variety of wound dressings are available and still widely used to treat such wounds [1,2,3,4,7,8,9,10]. Even though the properties of ultimate wound dressings are well identified, major difficulties arise when it comes to combining them within the same material [1,2,3,4,7,9]. In other words, realizing an optimal combination of desired properties in one wrapping material is still challenging, and therefore new materials with improved properties are highly anticipated. In addition, even though it is well known that metal ions such as Zn, Mg, Fe and Cu are necessary for efficient wound healing [11,12], their incorporation into dressings used for wound treatment was not attempted yet.

Recently, free-standing ultra-thin films (or so-called nanosheets, NSs) of biodegradable polymers (such as, e.g., poly(L-lactic acid), PLLA) have shown promise as efficient barrier covering wounds with any shape and protecting them for days without the need for daily changes [7,8,9,10,13]. The advantages of such NSs as wound wrapping material consist in: (i) relatively simple preparation procedure and easy thickness control; (ii) high surface adhesiveness and possibility to apply on surfaces with any shape; (iii) good air/gas exchange through such nano-scaled dressings and their biodegradability; (iv) excellent performance as barriers for infection and dirt, and so on [7,8,9,13]. In addition, wound repair sites sealed by such NSs during surgery were reported to show neither scars nor tissue adhesion [5,7,8,10].

Several metal ions are known to be of high importance and stimulate skin repair and recovery. Among them, metal ions of Mg, Cu, Zn and Fe were reported to play the most significant role [11,12]: Mg functions as a co-factor for enzymes involved in protein and collagen formation; Cu is a required co-factor for cytochrome oxidase, for cytosolic anti-oxidant superoxide dismutase, and for the optimal cross-linking of collagen; Zn is a co-factor for both RNA and DNA polymerase and its deficiency slows down wound healing significantly; and finally, Fe is required for the hydroxylation of proline and lysine, while its deficiency results in impaired collagen production [11,12]. Thus, loading NSs with nano-containers releasing the above metal ions is believed to stimulate faster skin regeneration/growth. This challenging task, upon successful realization, will certainly boost the use and market value of such NSs, also expanding significantly fields of their potential applications.

**Figure 1:**
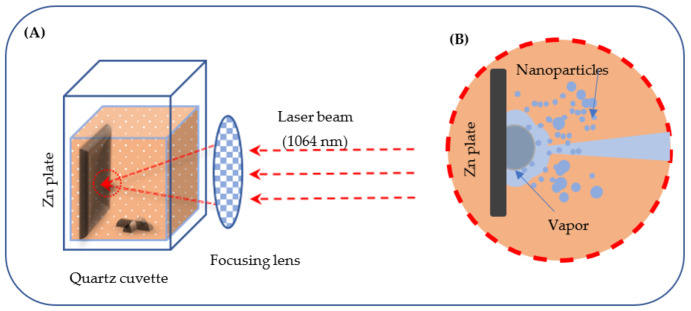
(**A**) Ablation of Zn plate immersed in chloroform with laser beam. (**B**) Nanoparticles forming in the ablation region and producing a brown colloid.

The laser ablation in liquid (LAL) is a quickly gaining in popularity and convenient laboratory technique to produce various nanostructures, including nanoparticles (NPs), nanocubes, hollow NPs, nanorods, nanoflakes and other structures of metals, metal oxides (see Figure 1), sulfides, carbides, etc. [14,15,16,17,18,19,20,21,22,23,24,25,26,27,28,29,30,31]. In this approach, laser beam is typically focused on a solid target, its pulses ablating the target and producing various nanostructures whose morphology, size and chemistry depend on the laser pulse parameters and liquid medium [14,15,16,18,19,20,21,25,29,30]. As a preparative method, LAL is attractive for several reasons: (i) it is an easy-to-use and green technique consuming minimum chemicals; (ii) a wide variety of nanostructures can be prepared, with control over their composition and morphology; (iii) the method operates at nominally room temperature while can often generate unique phases and morphologies. Importantly for the present study, LAL permits to prepare both Zn oxide and chloride NPs.

The overall goal of this work was to demonstrate that PLLA NSs can be embedded with NPs (as nanocontainers with Zn^2+^ ions) that can release metal ions when such NSs are placed on wet skin. Nanoparticles of ZnO and ZnCl_2_ were chosen as model NPs as those containing Zn^2+^ ions and having different solubility in water. Prior to incorporating PLLA NSs with multiple metal ions, Zn-containing NSs were chosen as a proof-of-concept system. By means of spin-coating, we first prepared NSs incorporated by Zn-containing NPs (both LAL-generated NPs and commercially available ones with similar composition), after which we tested how the as-prepared NSs released Zn ions after immersion in water at 37 °C and pH 7.4, i.e., conditions close to those on human skin. In addition, preliminary tests were carried out on LAL-prepared NPs to evaluate their ability to suppress *E. coli* bacteria. The obtained results will be of use for further development of PLLA nanosheets embedded with metal-ion-releasing nanocontainers as new generation biomedical materials (dressing for wound healing).

## 2. Methods and Materials

### 2.1. Materials

Zinc metal plate (99.5% purity) was purchased from Nilaco Corp. Chloroform (99.0% purity), deionized water and ethanol (99.5%) were purchased from Wako Pure Chemical Industries (Osaka, Japan). Phosphate-buffered saline solution (PBS) was purchased from Sigma-Aldrich (St. Louis, MI, USA), while polyvinyl alcohol, PVA was from Kanto Chemical Co. (Tokyo, Japan), and PLLA (Mw: 80–100 kDa) was supplied by Polysciences, Warrington, PA, USA. Zinc chloride salt was also purchased from Wako Pure Chemical Industries.

### 2.2. Nanoparticles

In this study, we prepared Zn-containing NPs by LAL of Zn in chloroform or water, aiming at obtaining ZnCl_2_ and ZnO NPs, respectively. While laser ablation of metallic Zn in water is known to result in zinc oxide or/and hydroxide NPs [15,17,18,23,26,28,29,31], LAL processing of Zn plate in chloroform was not reported before. Chloroform was chosen as reaction medium for LAL for two reasons: (i) because ZnCl_2_-based NPs were expected, and (ii) because chloroform was then used as solvent for spin-coating of PLLA NSs without the necessary to centrifuge produced NPs and redisperse them in chloroform.

A zinc metal plate with a thickness of 2.0 mm was cut into targets, 15 mm × 30 mm in size. The obtained plate was then immersed in ethanol and sonicated in an ultrasonic bath for 5 min to remove grease and surface contaminations. After drying in air for several minutes, the plate was fixed inside a quartz cuvette containing 15 mL of chloroform. Two types of Nd:YAG lasers with the fundamental wavelength of 1064 nm and either millisecond- or nanosecond-long pulses, were utilized for preparing nanomaterials. More details on setups and lasers used can be found elsewhere [17,18,30,31].

Chloroform used as liquid medium for LAL was stirred magnetically to disperse produced NPs throughout the ablation. As indicated in Table 1, experiments were conducted by using two lasers. The prepared colloids were then dropcast on Si wafers for X-ray photoelectron spectroscopy (XPS) analysis or on C-coated grids for transmission electron microscopy TEM.

### 2.3. Nanosheet Preparation

**Figure 2:**
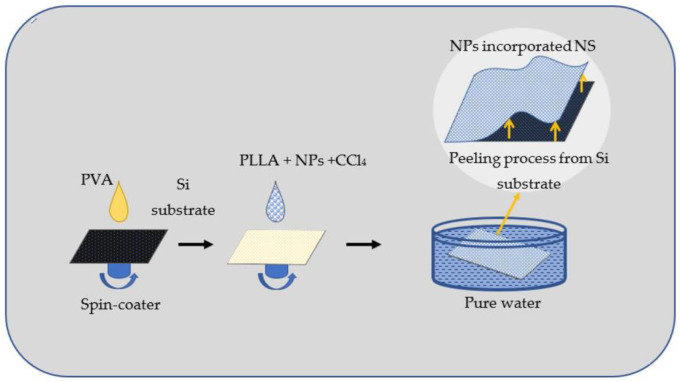
Preparation of nanoparticle-incorporated polymer nanosheets. PVA was used as sacrificial under-layer because of its solubility in water. When the formed PLLA/PVA structure is placed in water, the PLLA nanosheet begins to separate from Si substrate.

At first, 0.6 mL of PVA solution used as sacrificial film was dropped onto a silicon substrate (3 × 3 cm^2^), see Figure 2. The substrate was spin-coated at 4000 rpm for 20 s and then dried using a hot plate at 70 °C for 90 s. In parallel, 20 mg of PLLA in crystal form was added to 2 mL of a proper dispersion of NSs. At this stage, either commercial NPs or those produced by LAL were mixed with chloroform in a proper ratio (typically, 1.0, 2.0 or 4.0 mg/mL). The mixture was shaken using vortex mixer vigorously until PLLA crystals dissolved completely.

Then, 0.4 mL of the prepared mixture was spin-coated on the PVA-coated substrate. Again, the substrate was spun for 20 s and dried on a hot plate at 70 °C for 90 s. By using a cutter, a light cut was made on the prepared sample to make a NS with the size of 3 × 3 cm^2^. The substrate was then immersed in pure water (Figure 2), where it was moved from time to time using tweezers until the NS began to float. The NP-incorporated NSs were thus collected and transferred on polyester matts to preserve their shape.

### 2.4. Characterization

Morphology of obtained NPs and nanosheets were observed using scanning electron microscopy (SEM, S4800 from Hitachi, Tokyo, Japan) and transmission electron microscopy (HF-2200, Hitachi, Tokyo, Japan). Optical images of prepared NSs were observed by means of an SMZ 1500 (from Nikon, Tokyo, Japan) optical microscope. X-ray photoelectron spectroscopy (XPS) analysis was carried out on a PHI-1600 spectrometer (Physical Electronic Industries, Chanhassen, MN, USA), with all binding energies corrected for charge shifting by referencing to the adventitious carbon C1s line at 285 eV. Size of dispersed NPs was evaluated by means of dynamic light scattering (DLS) technique.

### 2.5. Ion Release in Buffered Saline Solution

The Zn^2+^ ion release from nanosheet samples was studied by means of inductively coupled plasma mass spectroscopy (ICP-MS). The samples were immersed in beakers with 40 mL of phosphate-buffered saline (PBS) solution (pH 7.4) at 37 °C. To determine the concentration of Zn^2+^ ions over time, aliquots of 1 mL were taken and analyzed after certain periods of time [32,33,34]. After each sampling, 1 mL of PBS solution was added to the sample. For each sample, experiments were carried out in triplicate, after which the obtained results were averaged.

To evaluate the antibacterial properties of incorporated nanoparticles, preliminary tests were conducted on LAL-generated NPs by pipetting them on a bacteria-coated medium and monitoring the growth of colony in the media by taking microscopic images using a laboratory microscope, as previously reported elsewhere [35].

## 3. Results and Discussion

### 3.1. Zn-Containing Nanoparticles

**Figure 3:**
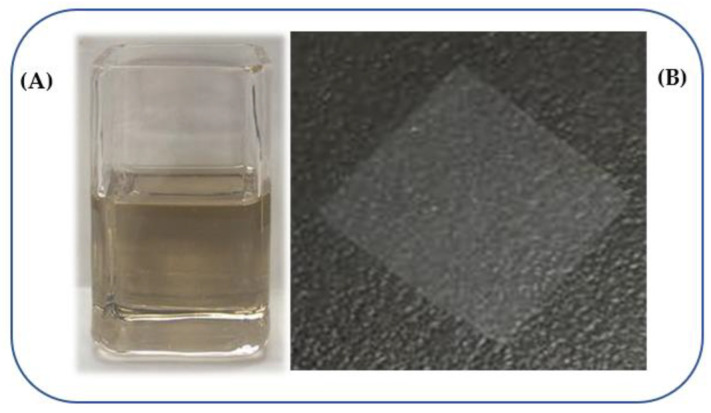
Cuvette with colloidal solution of Zn-Cl nanoparticles prepared via laser ablation in liquid (**A**) and free-standing PLLA nanosheet incorporated with such nanoparticles (**B**), floating on water. The nanosheet is 3 × 3 cm^2^ and about 50 nm thick, which provides superior flexibility and adhesion on any surface.

As a first step, we prepared various NPs via LAL, applying two different lasers (with millisecond- and nanosecond-long pulses) and using water or chloroform as liquid medium. Figure 3A presents a photograph of as-prepared colloid of Zn-based NPs produced via ablating Zn plate immersed in chloroform by means of ms-pulsed laser. Since chloroform was used as PLLA solvent during NS preparation, such homogeneous colloids with Zn-containing NPs were thus ready for further processing, i.e., for mixing with PLLA and spin-coating as NSs (see such a free-floating NS in Figure 3B).

**Figure 4:**
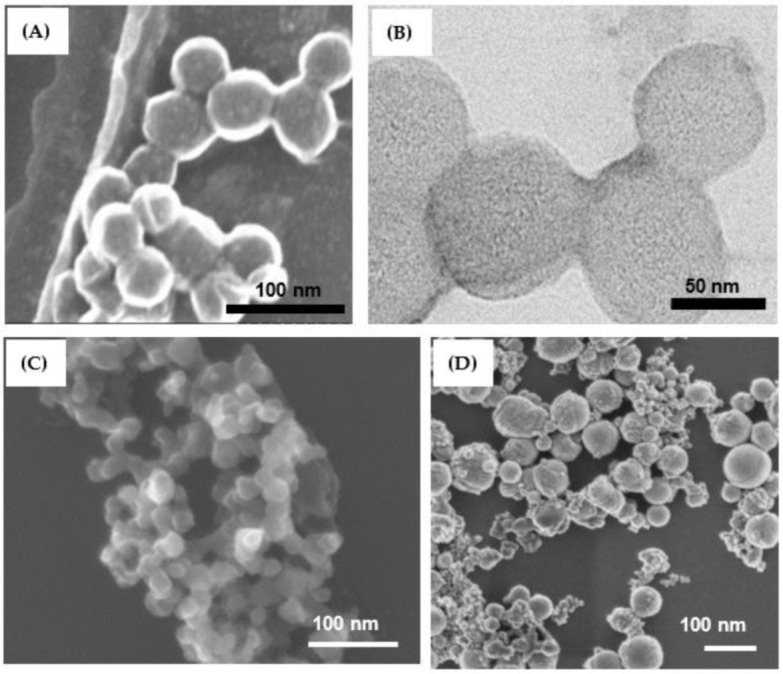
SEM (**A**) and TEM (**B**) images of Zn-Cl NPs prepared in chloroform by ms-laser. Pulse width, peak power and frequency used were 2 ms, 1 kW, and 10 Hz, respectively. SEM images of (**C**) commercial ZnO and (**D**) laser-produced ZnO NPs (ns-laser, water as medium).

**Figure 5:**
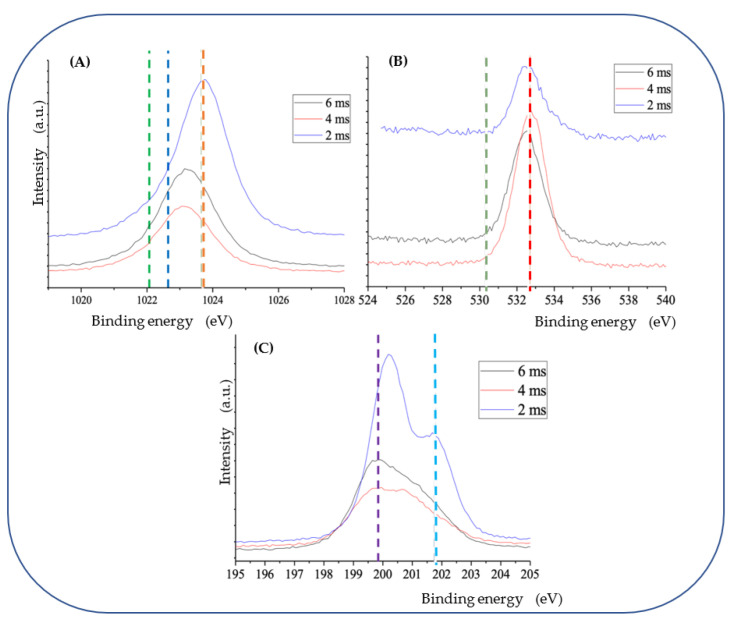
XPS spectra of Zn-Cl NPs produced with ms-laser in chloroform (pulse peak energy: 1 kW, varied pulse widths: 2, 4, 6 ms). (**A**) XPS Zn 2p_3/2_ spectra: metallic Zn (green dashed line); Zn-OH bonding (blue line); Zn-Cl bonding (orange line). (**B**) XPS O 1s spectra: O-C bonding (olive dashed line); OH species (red line). (**C**) XPS Cl 2p spectra: Zn-Cl bonding (purple dashed line); C-Cl bonding (light blue line).

Figure 4 presents NPs prepared by ms-laser in chloroform (A,B) and by ns-laser in water (D). The particles in panels A and B look quite uniform, being around 30–70 nm in size and showing rather amorphous morphology (Figure 4B). Images of other NPs prepared by ms-laser in chloroform using different conditions are shown in Supplementary Materials Figure S1, revealing quite similar morphology and sizes. As was revealed by XPS analysis (see Figure 5), NPs prepared by LAL of Zn in chloroform, along with dominating Zn-Cl species, also contained some fraction of surface Zn-OH, metallic Zn, and traces of C-Cl bonds [15,17,18,23,29,36]. Therefore, in contrast to NPs of commercial ZnCl_2_ salt, for simplicity in this study, we denoted such LAL-generated materials as Zn-Cl NPs. 

Note that other Zn-Cl NPs produced by both ms-laser and ns-laser in chloroform also had similar species as their components, even though, depending on conditions, such Zn-Cl NPs somewhat differed in chemical composition (see XPS spectra in Supplementary Materials Figure S2). The composition of LAL-generated Zn-Cl NPs was also confirmed by FTIR spectroscopy which compared their spectra with commercial ZnCl_2_ and ZnO products (see Supplementary Materials Figure S3). Thus, we confirmed that ablation of Zn in chloroform indeed led to NPs with ZnCl_2_ as the main phase. Changing laser parameters (see Figure S4), we could produce Zn-Cl NPs with slightly different sizes, generally within the range of ~30–150 nm. This is also confirmed by DLS results for two different Zn-Cl colloids presented in Figure S5, where the average size of NPs produced by ms-laser is seen to be around 55–60 nm.

Within the framework of the present study, no noticeable difference in Zn-ion release dynamics was observed between PLLA NSs incorporated with Zn-Cl NPs produced under different conditions (shown in Figure 4A,B and Figure S1). Hence, here, we only show representative SEM images (Figure 4A,B) and XPS spectra (Figure 5) of selected laser-produced Zn-Cl NPs, while more results are presented in Figures S1 and S2.

For comparison, as source of Zn^2+^ ions, we also aimed at using commercially available ZnCl_2_ NPs embedded into PLLA NSs. Since the salt was suppled in the form of relatively large particles (see Supplementary Materials Figures S6 and S7), we attempted to reduce their size by irradiating their dispersion in chloroform. It is clearly seen in Figures S6 and S7 that indeed irradiation of ZnCl_2_ particles suspended in chloroform led to gradual reduction in their average size. For instance, according to DLS analysis, irradiation for 40 min is seen in Figure S6 to permit to reduce ZnCl_2_ NPs to average sizes of around 120–140 nm. This size is already acceptable for embedding into NSs with average thickness of ~100 nm. 

In parallel, we also prepared ZnO NPs as their solubility in water is lower, and thus we expected slower Zn ion release from PLLA NSs incorporated with such particles. Commercial ZnO NPs with average size of ~20–25 nm (Figure 4C) and those produced using ns-laser (Figure 4D) were thus used to incorporate to PLLA NSs and test their Zn ion release. 

In addition, we also tested antibacterial properties of LAL-generated NPs, pipetting their dispersion on a bacteria-coated medium and monitoring the growth of colony in the media, following the methodology previously described elsewhere [35]. By taking optical microscopy images, we counted the number of colonies in presence and absence of laser-produced NPs, showing that the Zn-containing NPs we used inhibit the growth of *E. coli* to some extent (see Supplementary Materials, Figures S10 and S11). The obtained preliminary results imply that there might be some antibacterial effect when such NPs are incorporated into PLLA NSs and the latter NSs are applied on skin or wound. This is in line with reports of others on antibacterial effect of various ZnO NPs previously published elsewhere [26,29,37,38]. According to the literature, Zn^2+^ ions released by Zn-containing NPs could be one of factors suppressing certain bacteria strains [26,29,37,38].

### 3.2. Zn-Incorporated Polymer Nanosheets

**Figure 6:**
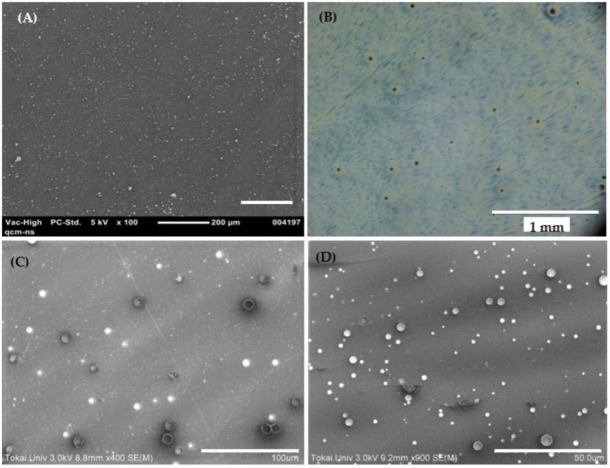
SEM images of PLLA NSs loaded with (**A**) commercial ZnO NPs, (**C**) Zn-Cl laser-generated NPs, and (**D**) commercial ZnCl_2_ NPs. (**B**) Optical image of nanosheet loaded with laser-generated ZnO NPs (ns-laser, water medium). Scale bar indicates 200 μm (**A**), 1 mm (**B**), 100 μm (**C**), and 50 μm (**D**).

As a second step, both the laser-produced and commercial ZnO NPs, as well as their counterparts based on ZnCl_2_, were incorporated into PLLA nanosheets. This was achieved via spin-coating mixtures of PLLA polymer and colloidal NPs in chloroform as solvent. Figure 6 shows surface images of PLLA NSs incorporated with commercial ZnO NPs (A), LAL-prepared ZnO NPs (B), commercial ZnCl_2_ NPs (C) and laser-prepared Zn-Cl NPs (D). 

Since the thickness of spin-coated PLLA NSs was around 100 nm, various Zn-containing NPs from tens of nm to 100–120 nm are seen in Figure 6 to be well incorporated. Even though we did not aim at optimizing spin-coating process and reach extremely homogeneous NP distribution, the conditions used permitted to achieve quite uniform particle distribution over the entire NS surface. Expectedly, the smallest ZnO NPs are seen in Figure 6A to be distributed better and with minimum agglomerates over the sample surface, while both samples with ZnCl_2_-based NPs are seen in panels C and D to demonstrate somewhat lower degree of uniformity over their surface, which is related to their larger NP size and wider size distribution.

We investigated the capacity of prepared PLLA NSs to incorporate ZnO NPs, which was done with commercial material. For this, NSs with 1, 2 and 4 mg/mL of ZnO powder (dispersed in PLLA solution in chloroform) were spin-coated. It was found that after more than 4 mg/mL of ZnO NPs, NSs prepared under the conditions used in this study were not mechanically strong and tended to disintegrate relatively easily. That is why, for ion release tests, NSs were prepared from ZnO dispersions in PLLA solution in chloroform with 1 and 2 mg/mL of ZnO.

For comparison, when preparing NSs incorporated with ZnCl_2_-based NPs, we loaded them with 3.3 mg/mL of ZnCl_2_ NPs, as this corresponds to the same amount of Zn ions as in 2 mg/mL of ZnO nanomaterial. However, it should be noted that during spin-coating the amount of incorporated nanomaterial depends on NP size, morphology and nature, given that the other parameters (such as PLLA concentration, chloroform as solvent and rotational speed) are fixed. Thus, embedding the same amount of Zn ions for different nanomaterials, even for ZnO NPs with different sizes, is quite difficult.

### 3.3. Ion Release from Prepared Polymer Nanosheets

**Figure 7:**
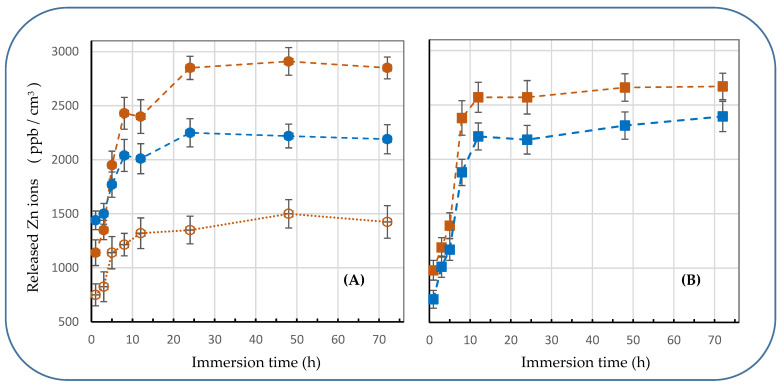
Concentration of Zn^2+^ ions released from PLLA NSs incorporated with ZnO NPs (**A**) and ZnCl_2_ NPs (**B**) over time into liquid with pH 7.4 at 37 °C. (**A**) Commercial ZnO NPs (orange) were loaded to spin-coated liquid at 2 mg/mL (solid circles) and 1 mg/mL (open circles), blue circles indicate NSs incorporated with LAL-generated ZnO NPs. (**B**) Results for PLLA NSs incorporated with commercial ZnCl_2_ (orange) and LAL-generated Zn-Cl NPs (blue) are presented (ms-pulsed laser was used). Dashed lines are only guide to the eye.

Figure 7 demonstrates how PLLA nanosheets embedded with different NPs released Zn^2+^ ions when immersed into physiological solution (pH 7.4). The solution simulated human body fluids and therefore was kept at 37 °C. Concentration of Zn ions released by 1 cm^2^ of NSs incorporated with ZnO NPs and ZnCl_2_ NPs is exhibited in panels (A) and (B), respectively. Except for one sample, which was incorporated with 1 mg/mL of commercial ZnO NPs (orange open circles, panel (A)), all the other NSs embedded with ZnO had either 2 mg/mL of ZnO (commercial NPs, orange solid circles) or 2 mg/mL of LAL-generated NPs (blue solid circles).

Comparison of the curves with orange and blue solid circles as markers (see Figure 7A) permits to conclude that both commercial and LAL-prepared ZnO NPs released Zn^2+^ ions in a similar way, with a similar slop and plateau reached after 12 to 20 h. Quite comparative level of maximum concentration was reached for NSs incorporated by ZnO with different sizes and size distribution. As mentioned above, the difference can be explained by a difference in the number and mass of ZnO particles spin-coated from different nanomaterials. Importantly, however, when same ZnO NPs were spin-coated from their dispersions with different concentration (1 mg/mL and 2 mg/mL of ZnO NPs, see orange empty and solid circles in Figure 7A), pretty good correlation was observed between the maximum concentrations achieved.

For PLLA NSs embedded with ZnCl_2_-based NPs (Figure 7B), again, similar results were demonstrated by samples with LAL-generated and commercial NPs (curves with blue and orange markers). The most interesting observation is that in both panels (A) and (B), maximum Zn^2+^ concentration is basically achieved ~10–12 h after immersion. This result is somewhat unexpected, taking into consideration the difference in water solubility between ZnO and ZnCl_2_. The similar dynamics of ion release observed in panels (A) and (B) (Figure 7) can probably be explained by (i) relatively small sizes of ZnO NPs, which accelerated their dissolution in water, and (ii) permeability of PLLA NSs as a limiting stage for Zn^2+^ ion release.

Importantly, it is clearly seen in Figure 7 that all the NSs incorporated with Zn-containing NPs released Zn ions actively within ~10 h, after which quite steady concentrations were reached. In light of this, the obtained results look very promising as dressing materials applied onto skin wounds are typically kept there for ~1 day and are expected to provide drugs or ions incorporated into them (as acting species) within a few hours upon their placement.

It should be noted that so far, there is no information yet on the desired concentrations of Zn^2+^ expected on skin surface for efficient healing, and this is still a subject for future investigations. Therefore, here, we only aimed at showing that concentration control of embedded Zn^2+^ ions is possible, and that embedded Zn-containing NPs can release ions within a short enough period of time. In parallel, the effect of developed Zn-incorporated NSs on healing of burn wounds on animals is also to be tested in the future. However, the fact that Zn ions are released from the developed polymer NSs as fast as within several hours shows promise. Obviously, wound dressings, when placed around wound area, should demonstrate their efficacy within several hours.

## 4. Conclusions

The study showed that biodegradable polymer nanosheets of poly(L-lactic acid), PLLA incorporated by Zn-containing nanoparticles can be prepared in a simple way by means of spin-coating. For this, commercially available and prepared via laser ablation in liquid ZnO and ZnCl_2_ nanoparticles were used. We showed that PLLA nanosheets can be embedded by different Zn-containing nanoparticles, with different loading and with reasonably good (homogeneous) distribution of nanoparticles across entire nanosheet. After preparation, the nanosheets were immersed in a buffered liquid with pH 7.4 at 37 °C, where their Zn ion release was monitored over time. Interestingly, irrespective of the nature of Zn-containing nanoparticles, the dynamics of their Zn ion release was quite similar, with maximum concentration achieved before ~10 h. This finding is very promising for further optimization and development of convenient wound patches based on such inexpensive biodegradable nanosheets, as release of incorporated metal ions within several hours after such patches are applied on skin is of high importance.

## Figures and Tables

**Table 1:** Laser parameters used in experiments.

	Ns-Pulsed Laser	Ms-Pulsed Laser
Laser output	40, 60, 80 mJ/pulse	2, 4, 6 kW
Pulse width	5–7 ns	2, 4, 6 ms
Frequency	10 Hz	10 Hz
Ablation time	15 min	15 min

## Data Availability

Not applicable.

## Supplementary Materials

The following supporting information can be downloaded at: https://www.mdpi.com/article/10.3390/ma15228101/s1. Figure S1: SEM images of laser-prepared Zn-Cl nanoparticles; Figure S2: XPS spectra of laser-prepared Zn-Cl samples; Figure S3: FTIR spectra of samples; Figure S4: Size of Zn-Cl nanoparticles as a function of laser parameters used; Figure S5: Histograms with size distribution of laser-prepared Zn-Cl nanoparticles; Figure S6: Size of commercial ZnCl_2_ particles irradiated with laser in acetone; Figure S7: SEM images of ZnCl_2_ before and after irradiation; Figure S8: Schematic presentation of antibacterial test used; Figure S9: Schematic presentation of bacteria colonies counted around nanoparticles; Figure S10: Colonies of *E. coli* formed around different nanoparticles; Figure S11: Number of colonies counted around different nanoparticles.
