# Supplementary material for: Biodegradable Polymer Nanosheets Incorporated with Zn-Containing Nanoparticles for Biomedical Applications
Source: Materials (Basel). 2022 Nov 16;15(22):8101. doi: 10.3390/ma15228101 (PMC9694524; doi:10.3390/ma15228101)
Supplement: Supplementary file 1 [file materials-15-08101-s001.zip › materials-2009553-supplementary.pdf]

Supplementary Material for

Biodegradable Polymer Nanosheets Incorporated with Zn-Containing Nanoparticles for Biomedical Applications

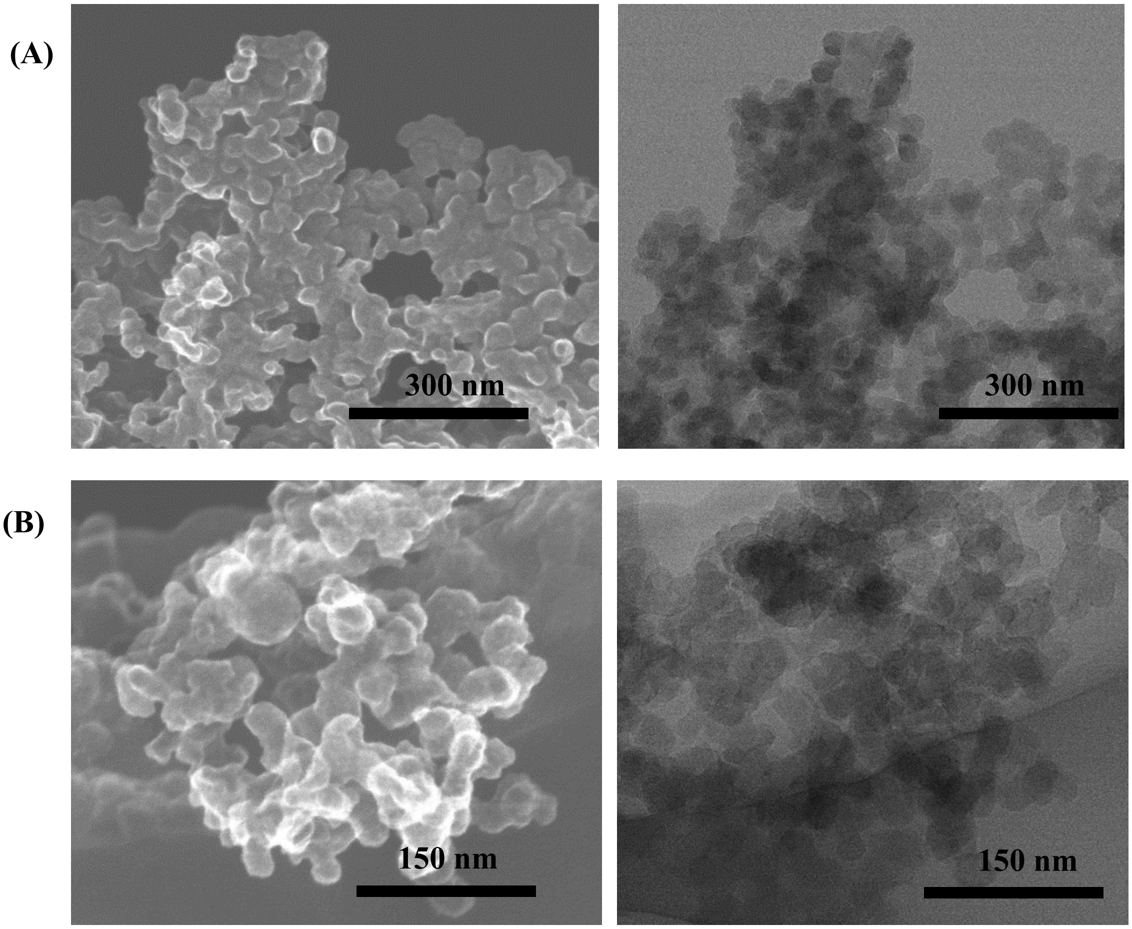

Figure S1. Microscopic images of Zn-Cl nanoparticles prepared by ms-pulsed laser in chloroform using different laser parameters. The images were taken by electron microscopy in different modes (left: SEM, right: TEM). (A) 1 kW, 0.5 ms and 10 Hz as pulse peak energy, pulse width and frequency, respectively. (B) 4 kW, 0.5 ms and 10 Hz as pulse peak energy, pulse width and frequency, respectively.

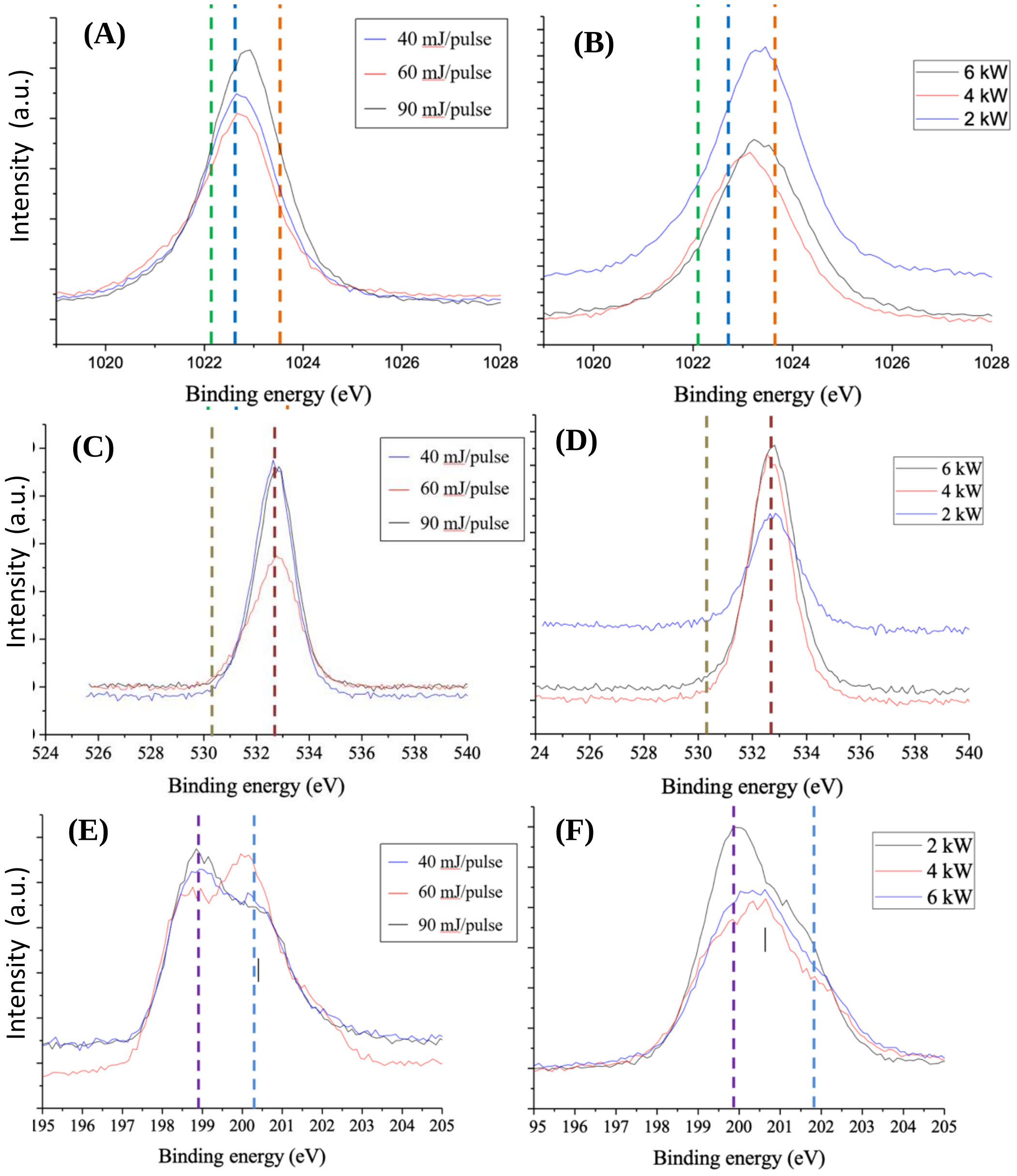

Figure S2. XPS spectra of Zn-Cl NPs produced with ns-laser (A,C,E) using different pulse energy and ms-laser (B,D,F) using different pulse peak energy in chloroform. (A,B) XPS Zn $2p_{3/2}$ spectra: metallic Zn (green dashed line); Zn-OH bonding (blue line); Zn-Cl bonding (orange line). (C,D) XPS O 1s spectra: O-C bonding (olive dashed line); OH species (red line). (E,F) XPS Cl 2p spectra: Zn-Cl bonding (purple dashed line); C-Cl bonding (light blue line).

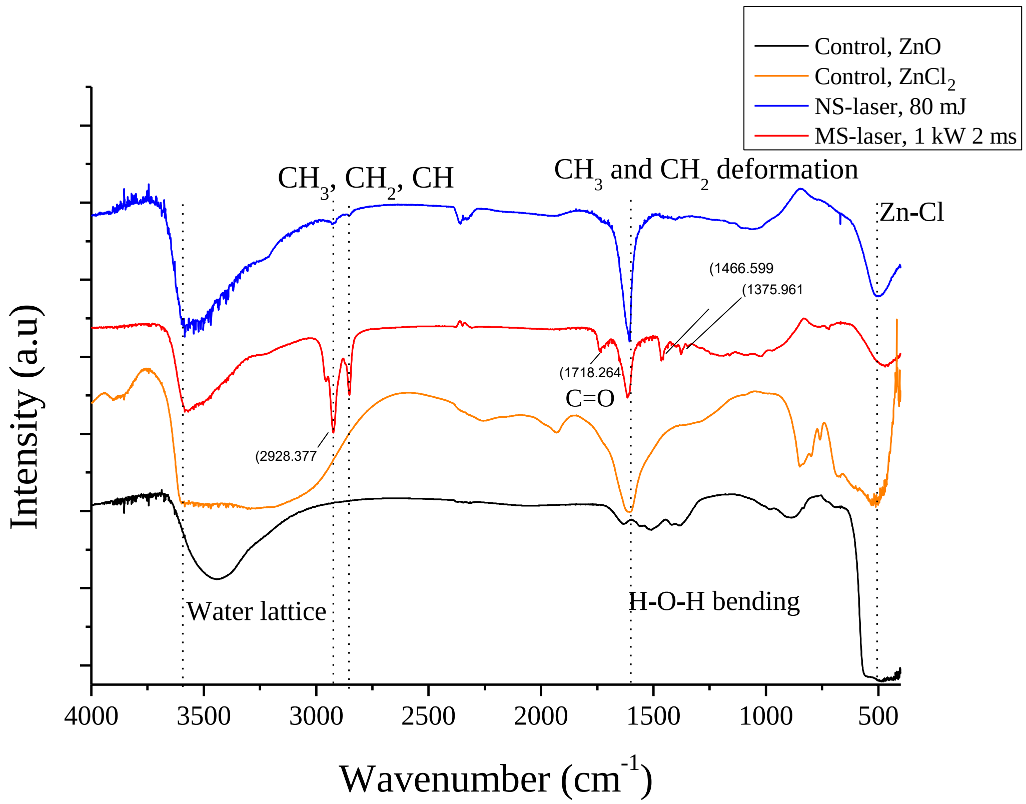

Figure S3. FTIR spectra of samples prepared using ns- and ms-pulsed lasers in chloroform. Commercial $ZnCl_2$ and ZnO nanoparticles (orange and black curves) were also measured for reference.

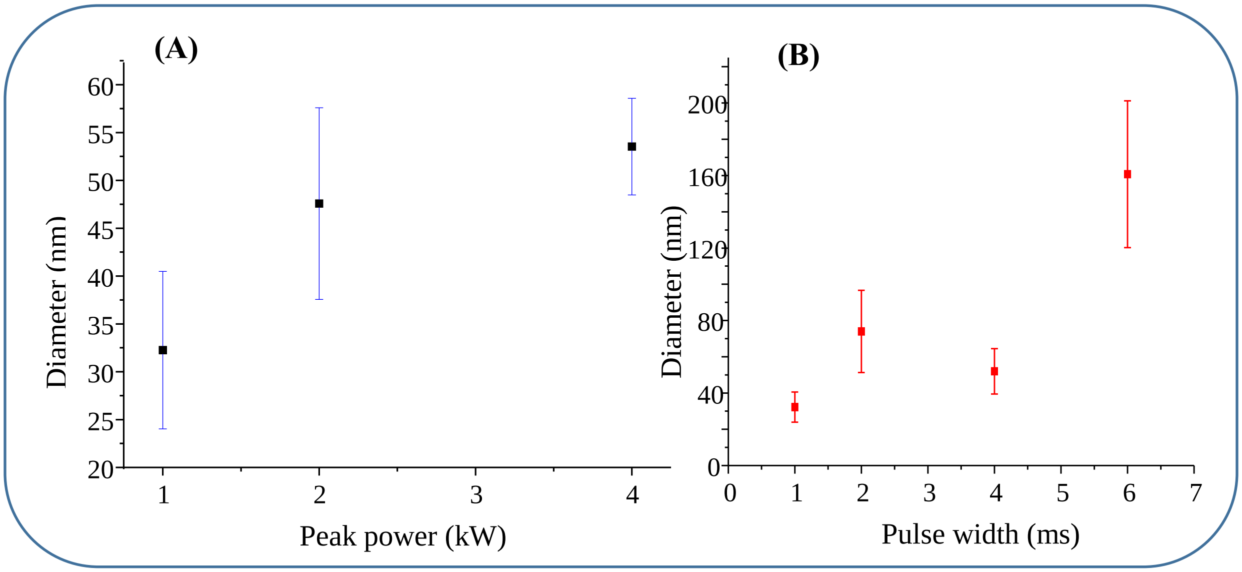

Figure S4. Effect of laser peak power (A) and pulse width (B) on NP size of material prepared by ablating Zn in chloroform. Pulse width was fixed at 1 ms (A) or laser peak power was kept to 1kW (B).

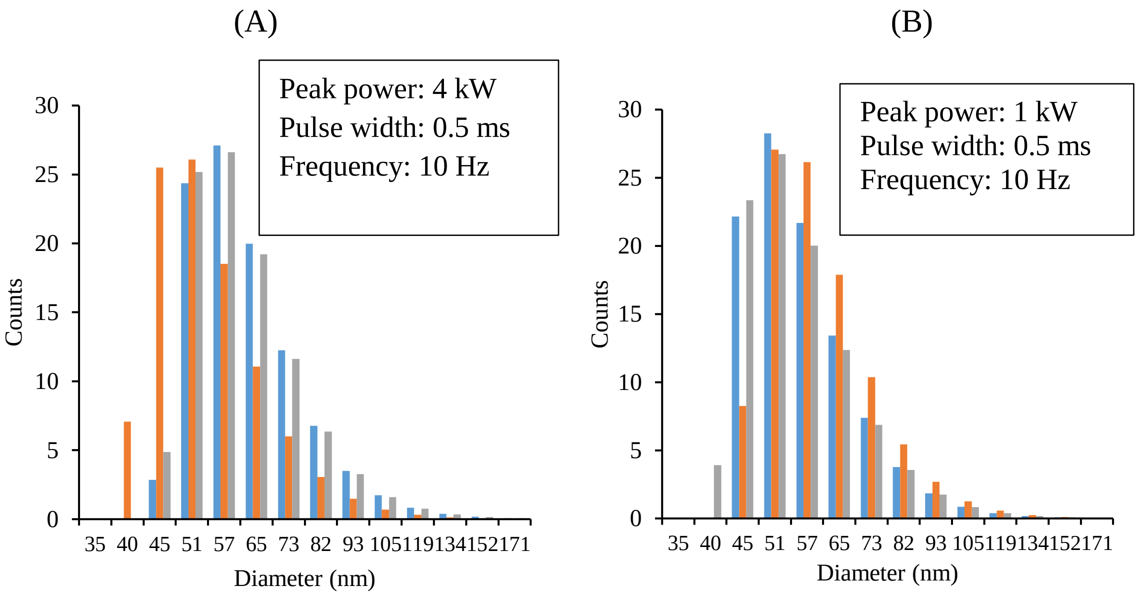

Figure S5. DLS of samples prepared by ms-pulsed laser in chloroform at different laser power. Orange, blue and grey colors present results of three subsequent measurements.

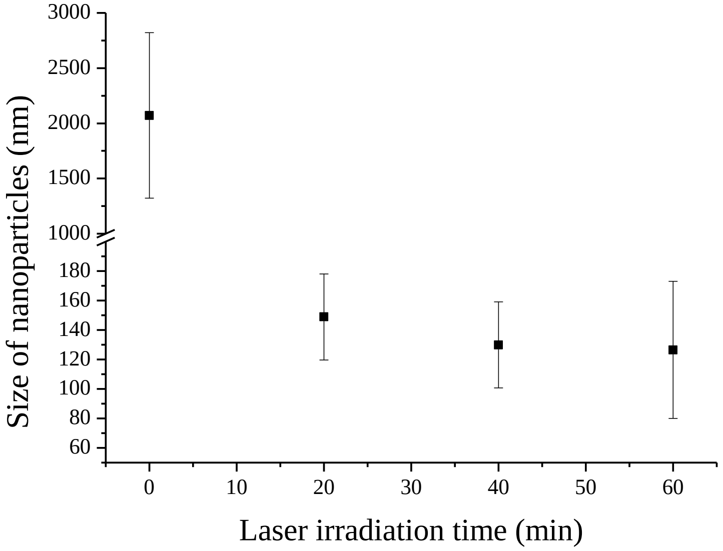

Figure S6. Size of laser-irradiated Zn-Cl as a function of irradiation time (as evaluated by DLS in acetone).

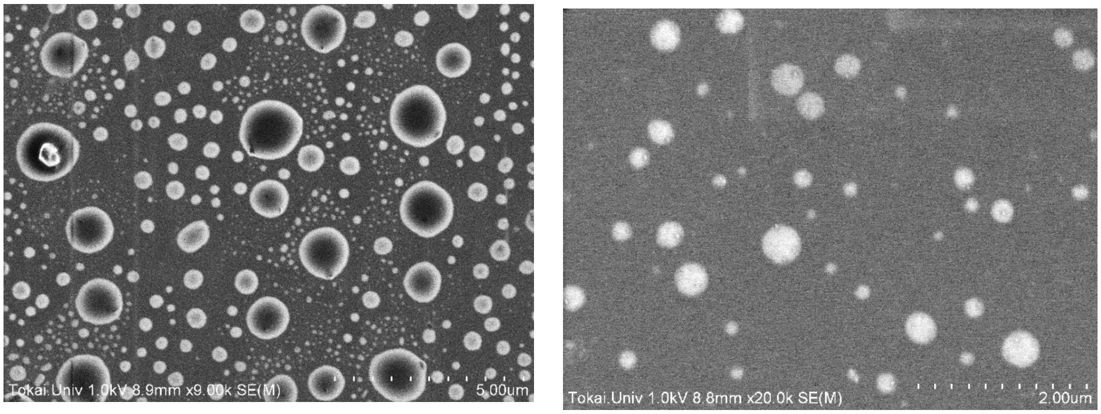

Figure S7. Morphology of commercial $ZnCl_2$ nanoparticles on Si substrate before (left) and after (right) irradiation at 4 kW laser energy.

Antibacterial tests of LAL-generated nanoparticles

Bacteria Cultivation

Escherichia coli (DH1) bacteria were inoculated on a Luria-Bertani (Wako Pure Chemical Industries, Ltd.) culture medium with a platinum loop and incubated at 37°C for 24 h to form colonies. After 24 h, the growth of colonies was observed, and the colonies were taken for antibacterial tests. By following the McFarland turbidimetric method, amount of bacteria in a solution can be determined. A platinum loop was used to transfer colonies of DH1 from the cultivated culture medium to a new liquid culture medium. After that, the mixture was stirred with a vortex mixer, and the absorbance intensity was taken around 0.699 a.u using an ultraviolet-visible spectrophotometer (UV-2450, Shimadzu) to adjust the concentration of DH1 to McFarland 4.0. At McFarland 4.0, the amount of colony in each mL of the liquid medium was 12×10^{8} CFU (CFU, colony forming unit) giving the theoretical amount of DH1 as 6000×10^{4} units [*J. Microbiol. Methods* **2016**, *122*, 59-63].

The solution of DH1, which was previously adjusted to McFarland 4.0, was spread on a new LB culture media using a steel spreader in a lattice pattern. The *E. coli* coated medium was used in antibacterial tests of $ZnCl_2$ nanoparticles and polymer nanosheets incorporated with nanoparticles. All procedures were conducted in a clean-bench with UV-lamp to avoid contamination. Sterilization of tools was done in an auto-clave. Platinum loop and steel spreader were sterilized by flame each time before use.

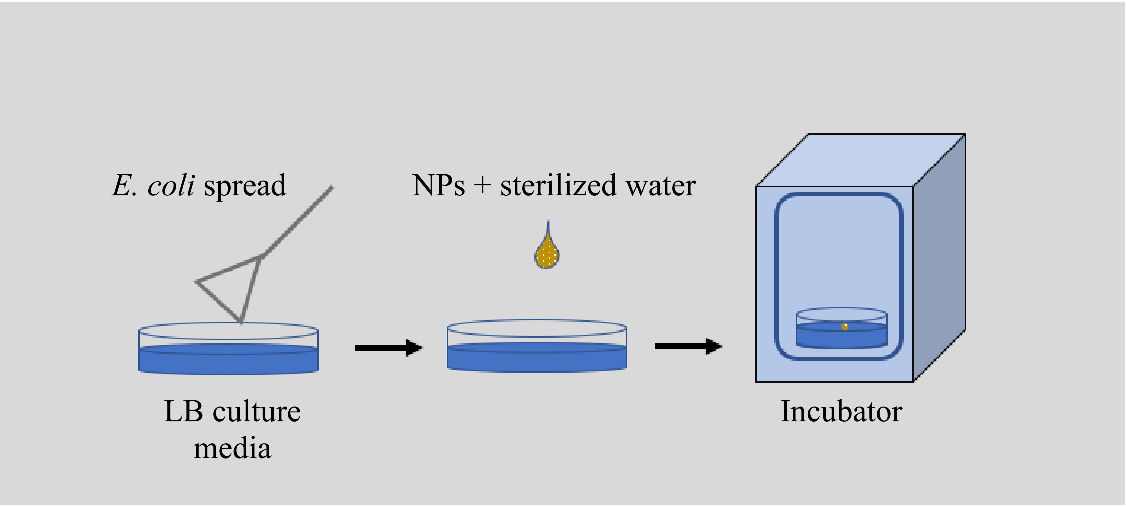

Figure S8. Schematic presentation of antibacterial tests of nanoparticles generated by ms and ns-pulsed lasers.

Colloids with $ZnCl_2$ or ZnO nanoparticles produced by LAL were transferred into a centrifuging tube and dried at normal temperature to remove chloroform residue. The amount of transferred material was evaluated through the loss of ablated target. After drying for 1 h, 50 μL of sterilized pure water was pipetted into the tube and sonicated for 30 min. The solution was dropped carefully on a bacterial-coated media and incubated for two days. Measurement of the antibacterial effect of the nanoparticles was taken by counting the number of colonies surrounding the location of nanoparticles, as shown in Figs. S8 and S9.

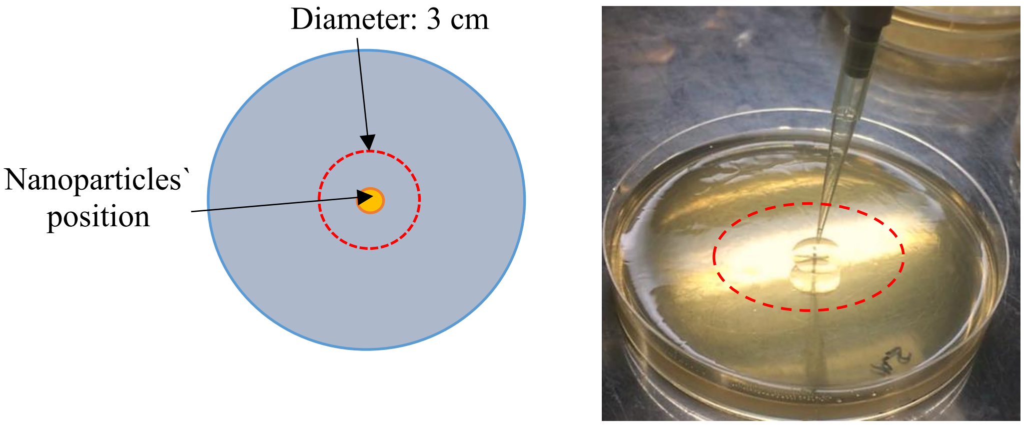

Figure S9. Number of colonies around nanoparticles was dropped were calculated using a smartphone application (Colony Count).

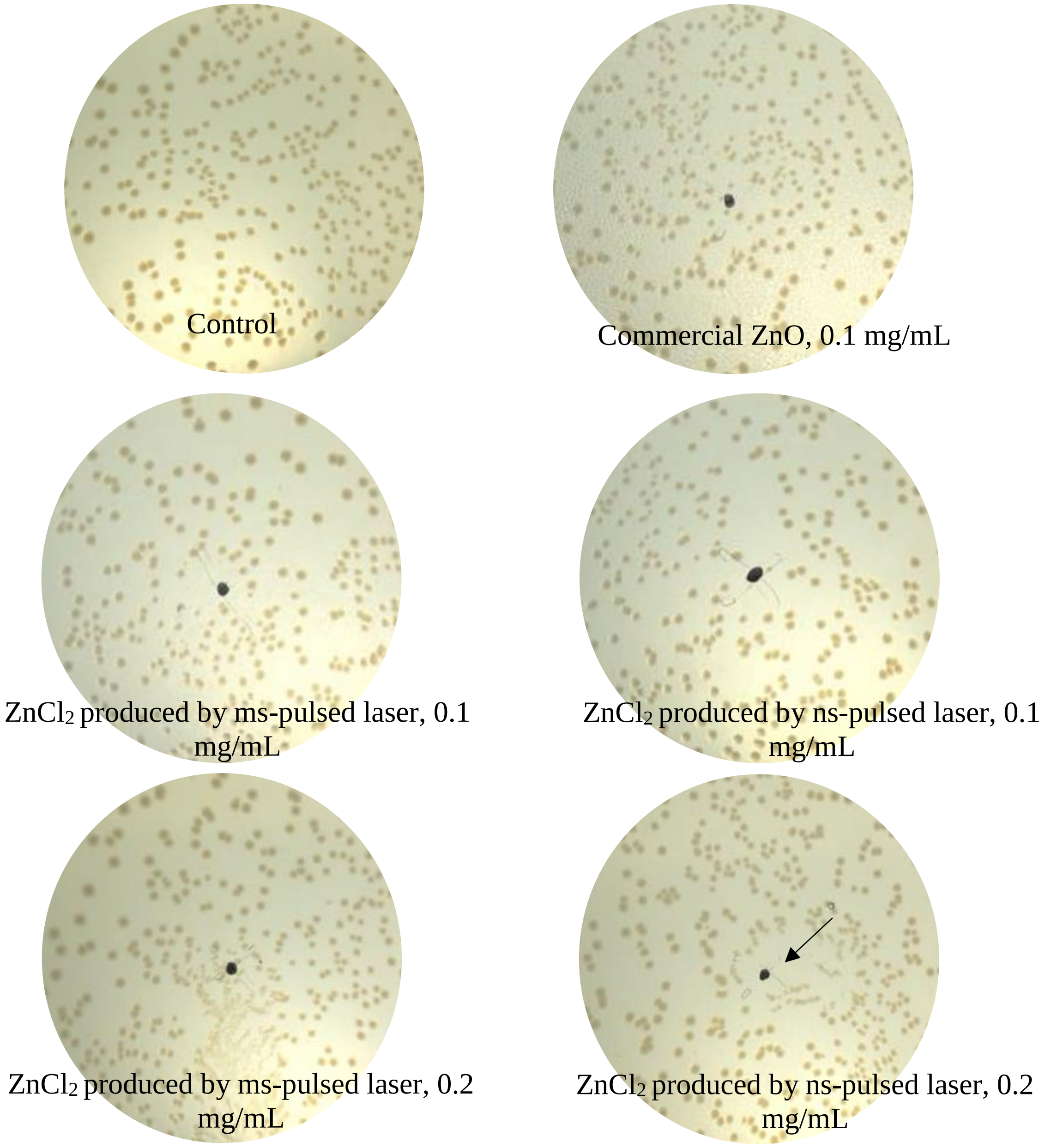

Figure S10. Colonies of *E. coli* formed on culture media. Black dot marks the position where solution with nanoparticles was dropped.

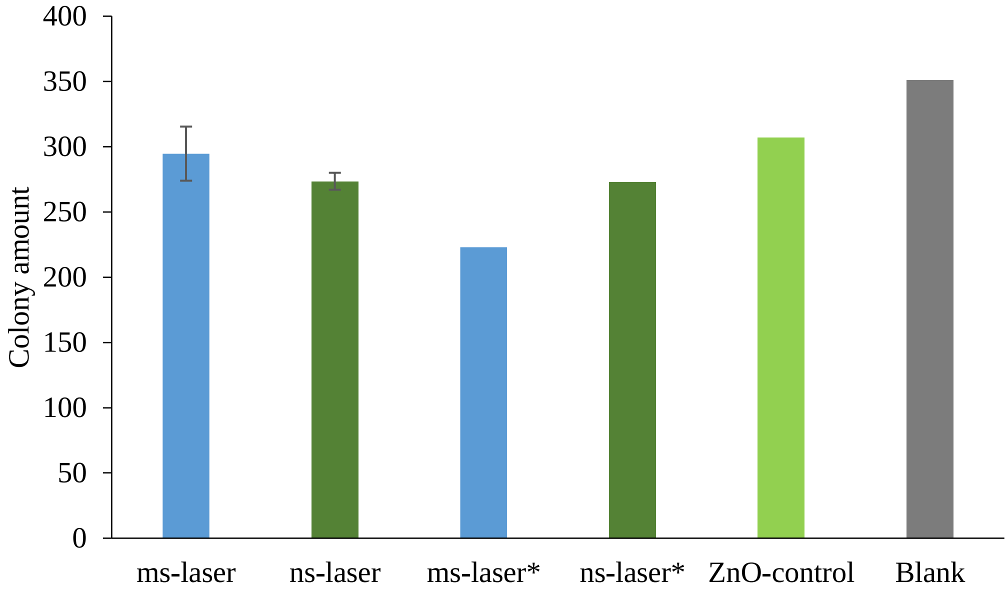

Figure S11. Number of colonies counted by Colony Count in area around position where nanoparticles were placed. Samples with * mark had 0.2 mg/mL. The other samples had 0.1 mg/mL.

The bacterial colonies inside culture media and in contact with commercial ZnO nanoparticles were found to be smaller compared to control sample. In the sample with $ZnCl_2$ prepared by ns-pulsed laser (0.2 mg/mL), there are fewer colonies around the nanoparticles` location compared to the samples prepared by ms-pulsed laser (see Figs.S10 and S11). There are many factors that can be associated with the effect of nanoparticles on the growth of the bacterial colonies. In a controlled experiment (controlled concentration of nanoparticles), we confirmed the antibacterial effect of $ZnCl_2$ on limiting the growth of bacterial colony. Spreading technique of bacteria in preparation process highly influenced the colony size as well.
